# Supplementary material for: Activation of neuronal genes via LINE-1 elements upon global DNA demethylation in human neural progenitors
Source: Nat Commun. 2019 Jul 18;10:3182. doi: 10.1038/s41467-019-11150-8 (PMC6639357; doi:10.1038/s41467-019-11150-8)
Supplement: Supplementary file 1 — Supplementary Information [file 41467_2019_11150_MOESM1_ESM.pdf]

Supplementary Information

Activation of neuronal genes via LINE-1 elements upon global DNA demethylation in human neural progenitors

Jonsson et al

Supplementary Figure 1

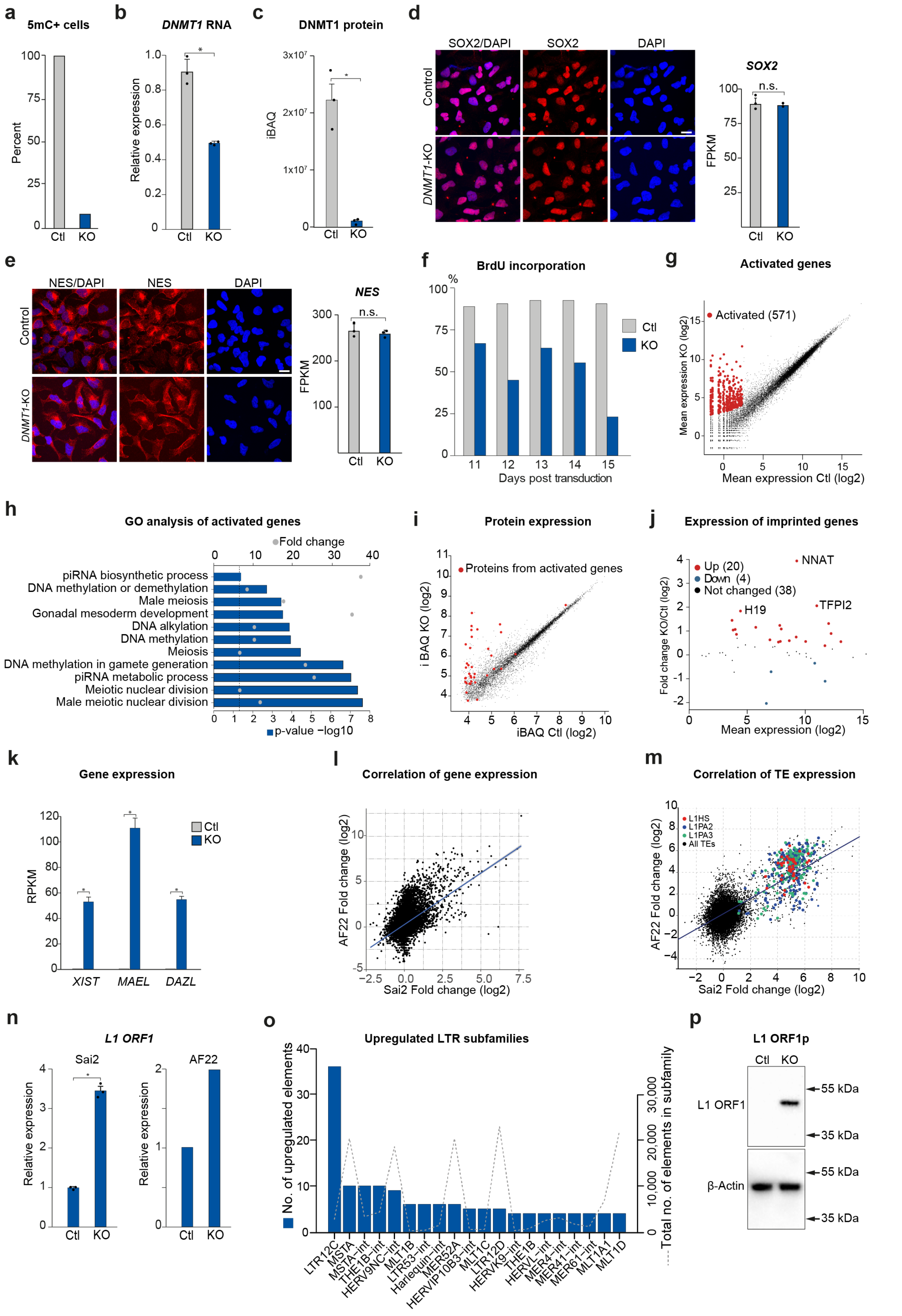

Supplementary Figure 1.

(a) The number of cells expressing 5mC in the expanding hNPCs was 99.1% vs 8.0% in the *DNMT1*-KO hNPCs as quantified by high content screening, Cellomics™. (b) qRT-PCR for *DNMT1* revealed a reduction but not a complete loss of the transcript in the *DNMT1*-KO hNPCs. (c) Mass spectrometry revealed a strong downregulation of the DNMT1 protein in the *DNMT1*-KO hNPCs, $p<0.05$ Student's t-test. (d-e) The expression of the hNPC markers SOX2 and NESTIN did not change upon *DNMT1*-KO as shown by fluorescent immunostaining post FACS at day 10, scalebar 25 µm, and qRT-PCR, Wald test, n=3 in both groups. (f) Following FACS at day 10, BrdU incorporation was monitored at 5 consecutive days and cells were quantified by high content screening, Cellomics™. (g) RNA-seq analysis detected activation of several protein coding genes upon the *DNMT1*-KO (n=3), BH-corrected p-value < 0.05. (h) A GO term overrepresentation test was performed on activated genes using Panther BP (the top 11 terms are listed). The p-values are Bonferroni-corrected. (i) The proteomic analysis detected several proteins from the activated genes found in the RNA-seq analysis. Dots are mean values of triplicates, where red dots show protein products of genes significantly activated in RNA-seq data and black dots show all detected proteins in the mass-spectrometry. (j-k) The expression of imprinted genes and XIST upon loss of DNA methylation, as analyzed by RNAseq, BH-corrected p-value < 0.01. n=3 in both groups. (l) The correlation of gene expression upon *DNMT1*-KO in two different hNPC cell lines, Sai2 (Tailor et al., 2013) n=3, and AF22 (Falk et al., 2012), triplicates pooled prior to FACS into n=1 (m) The correlation of TE expression in the two hNPC cell lines showed that the upregulation of evolutionary young L1s upon *DNMT1*-KO had a strong correlation between the two cell lines. Both X and Y chromosomes were excluded from the analysis. (n) The upregulation of L1 expression was verified by qRT-PCR in both cell lines. (o) The average number of upregulated elements in each LTR family upon *DNMT1*-KO and the total number of elements in each family are plotted on the left and right y-axes, respectively (n=3 in both groups). (p) Western blot for L1 ORF1p (42kDA) revealed the presence in *DNMT1*-KO cells and not in Control cells.
Raw data for b, c, d, e, k, n, p in this figure is provided as a Source Data file.

Supplementary Figure 2

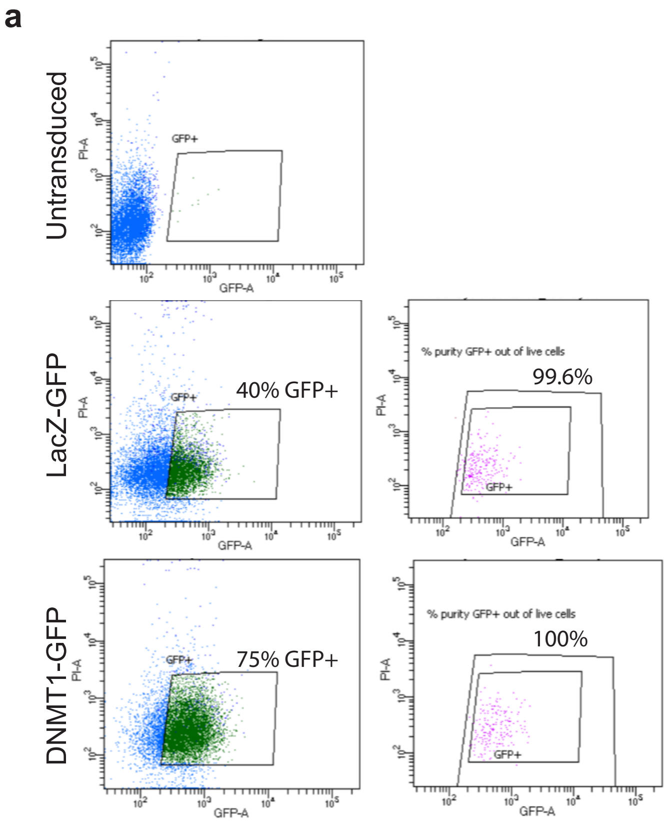

Supplementary Figure 2.
(a) FACS of Sai cells 10 days post transduction with LV.LacZ and LV.DNMT1-CRISPR vectors. GFP gates were set based on untransduced cells and the GFP expressing control and *DNMT1*-KO cells were sorted to a purity close to 100 %.

Supplementary Figure 3

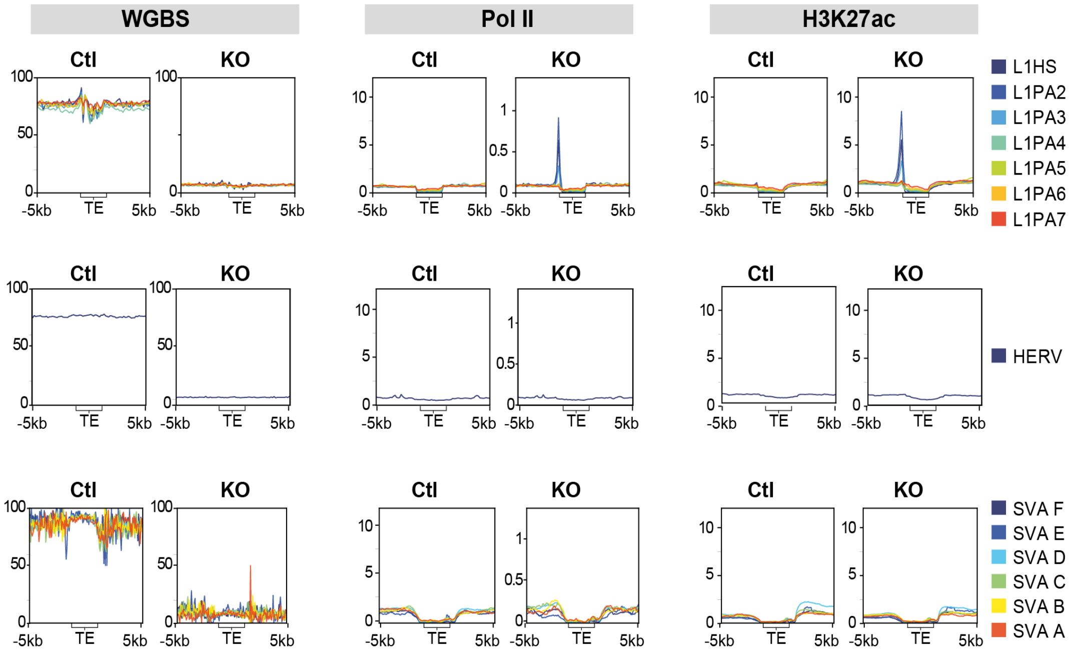

b

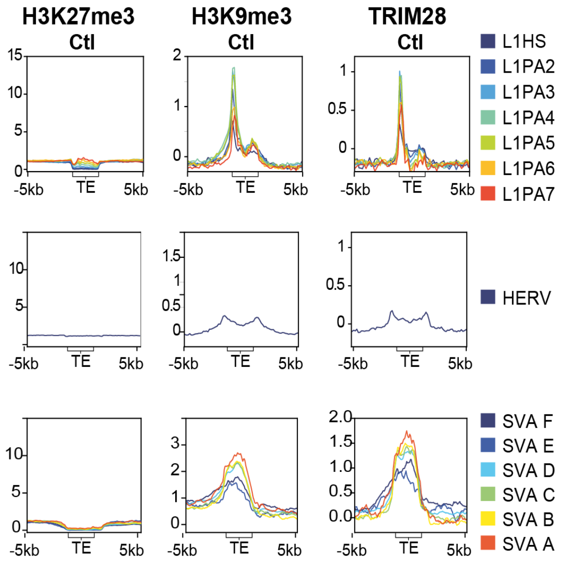

Supplementary figure 3.

(a) Histograms related to fig. 2i of WGBS, ChIP-seq of Pol II and H3K27ac in control and *DNMT1*-KO hNPCs in the eight youngest L1 subfamilies, all FL-HERVs, and all SVAs. WGBS data are shown as % mCpG at all CpG sites. ChIP-seq of Pol II and H3K27ac is shown as mean signal of both replicates at each base. (b) Histograms related to fig. 2j of ChIP-seq signals of TRIM28, H3K9me3, and H3K27me3 in hNPCs in the eight youngest L1s, FL-HERVs, and SVAs. ChIP-seq of TRIM28 and H3K9me3 is shown as log2ratio of IP vs input. ChIP-seq of H3K27me3 is shown as mean signal of two replicates at each base.

Supplementary Figure 4

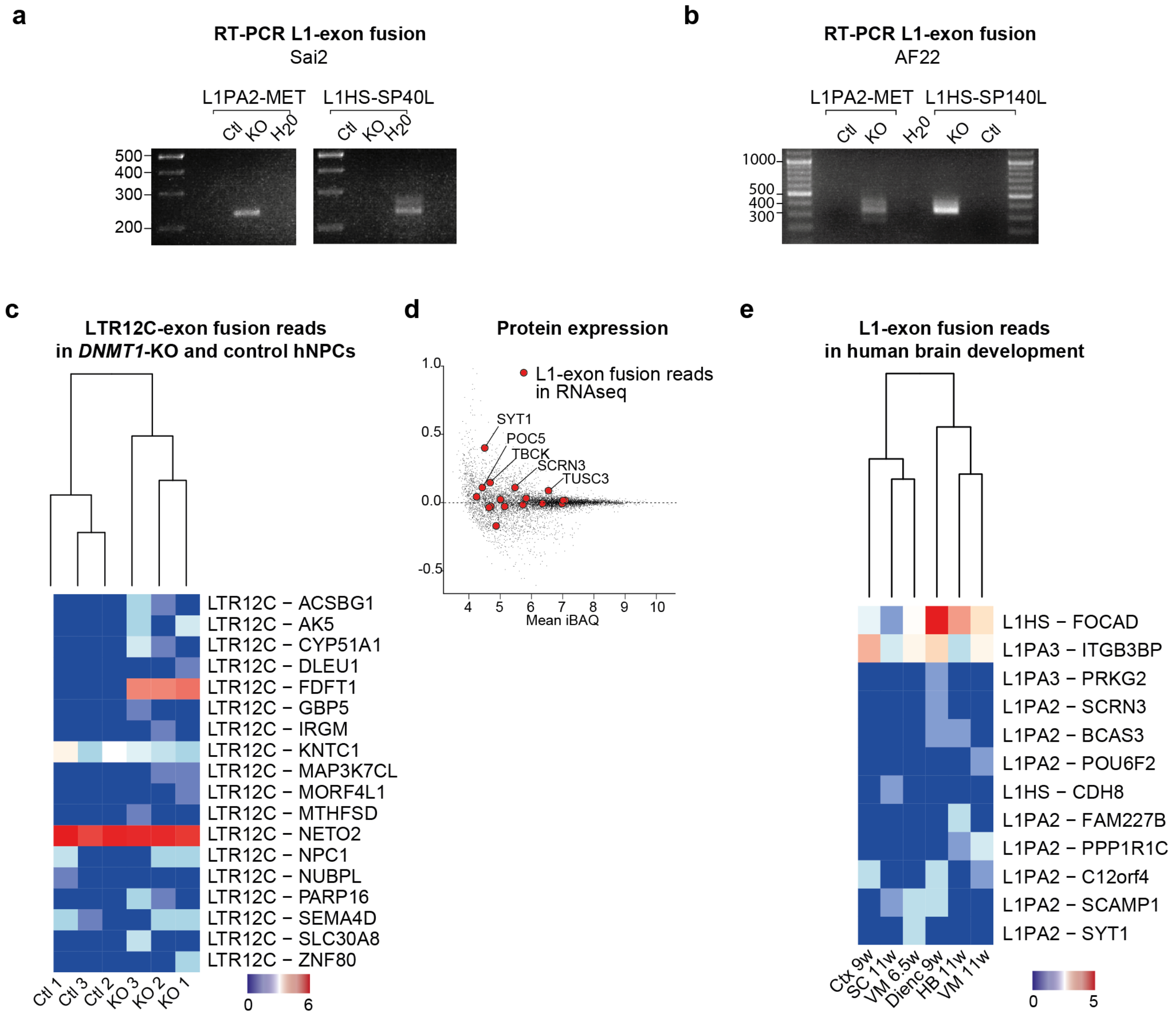

Supplementary figure 4.
(a) The presence of two L1-gene fusion transcripts 10 days upon *DNMT1*-KO were validated by RT-PCR of cDNA from ctl and *DNMT1*-KO Sai2 cells, (b) as well as in the additional hNPC line investigated, AF22. One primer recognized the L1 element and the other an exon in the gene, see Supplementary Table 4. (c) A heatmap showing the relative expression of LTR-exon fusion reads in three biological replicates of *DNMT1*-KO and control hNPCs. Only LTR-exon fusion reads for genes in which the LTR overlap annotated TSS are included. (d) Mass spectrometry detected several proteins in all triplicates from genes of which L1-exon fusion reads were found in the RNA-seq analysis of *DNMT1*-KO hNPCs. (e) Several L1-fusion transcripts were detected at different ages and in different regions of the developing human brain. Ctx – cortex, SC – spinal cord, VM – ventral mesencephalon, Dienc – diencephalon, HB – hindbrain.

Supplementary Figure 5

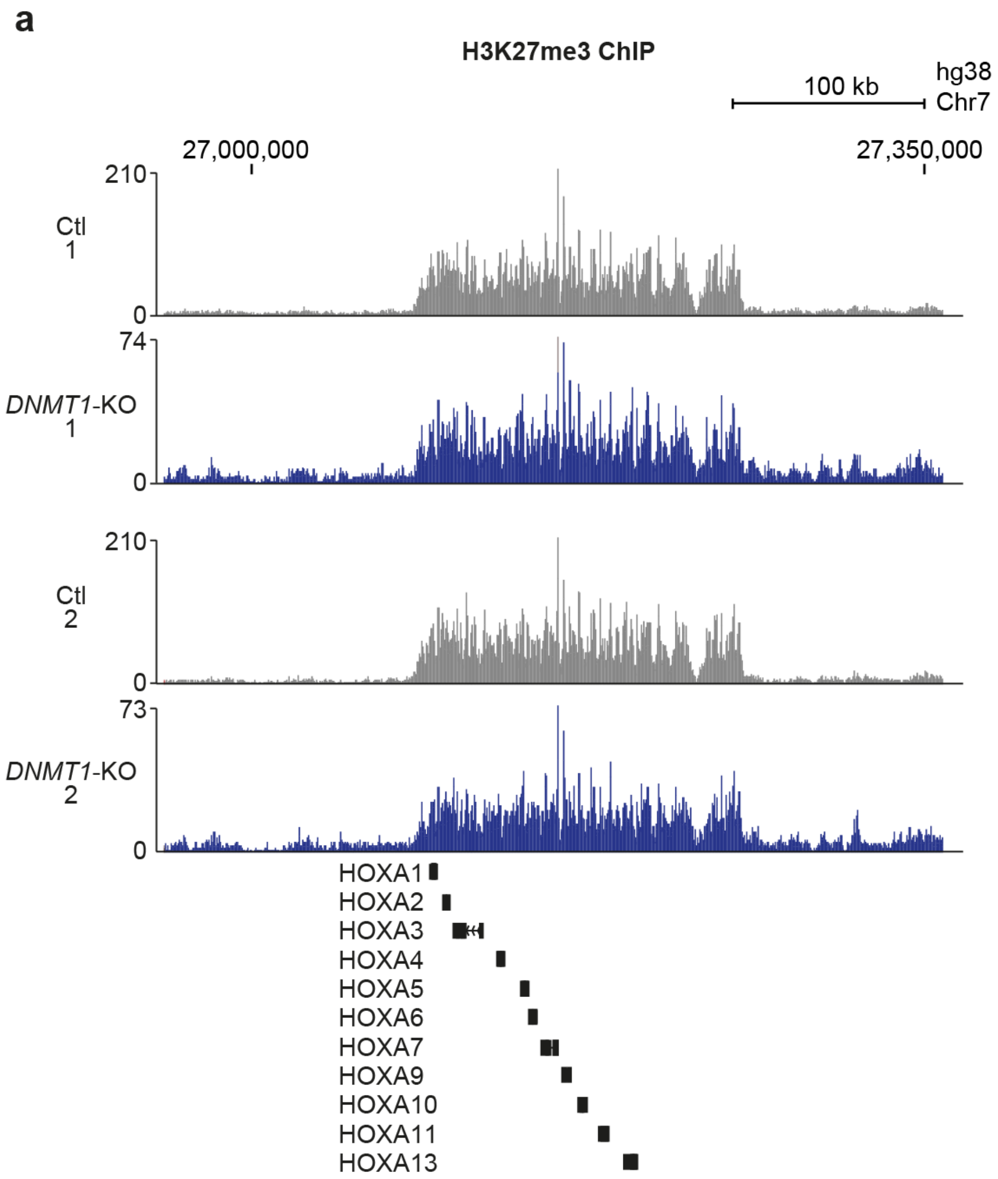

Supplementary figure 5.
(a) As a positive control for the H3K27me3 ChIP, USCS screen shots of the HOXA gene cluster on chromosome 7 showed strong H3K27me3 signals.
