# Supplementary material for: Activation of neuronal genes via LINE-1 elements upon global DNA demethylation in human neural progenitors
Source: Nat Commun. 2019 Jul 18;10:3182. doi: 10.1038/s41467-019-11150-8 (PMC6639357; doi:10.1038/s41467-019-11150-8)
Supplement: Supplementary file 3 — Description of Additional Supplementary Files [file 41467_2019_11150_MOESM3_ESM.pdf]

Description of Additional Supplementary Files

File name: Supplementary Data 1

Description: A list of the activated genes in the *DNMT1*-KO vs control NPCs.

File name: Supplementary Data 2

Description: We found 131 primate-specific L1s with potential alternative promoter activity which are listed in this table. These L1s were identified by their overlap with TSS coordinates of protein-coding genes in gencode (v25).

File name: Supplementary Data 3

Description: A complete list of GO terms upon analysis of genes influenced by L1 activation.

File name: Supplementary Data 4

Description: All primers used in this study are listed in this table.
