# Supplementary material for: Activation of neuronal genes via LINE-1 elements upon global DNA demethylation in human neural progenitors
Source: Nat Commun. 2019 Jul 18;10:3182. doi: 10.1038/s41467-019-11150-8 (PMC6639357; doi:10.1038/s41467-019-11150-8)
Supplement: Supplementary file 8 — Reporting Summary [file 41467_2019_11150_MOESM8_ESM.pdf]

nature research

Corresponding author(s): Johan Jakobsson

Last updated by author(s): Jun 3, 2019

Reporting Summary

Nature Research wishes to improve the reproducibility of the work that we publish. This form provides structure for consistency and transparency in reporting. For further information on Nature Research policies, see Authors & Referees and the Editorial Policy Checklist.

Statistics

For all statistical analyses, confirm that the following items are present in the figure legend, table legend, main text, or Methods section.

n/a	Confirmed	
☐	☒	The exact sample size (*n*) for each experimental group/condition, given as a discrete number and unit of measurement
☐	☒	A statement on whether measurements were taken from distinct samples or whether the same sample was measured repeatedly
☐	☒	The statistical test(s) used AND whether they are one- or two-sided<br>*Only common tests should be described solely by name; describe more complex techniques in the Methods section.*
☐	☒	A description of all covariates tested
☐	☒	A description of any assumptions or corrections, such as tests of normality and adjustment for multiple comparisons
☐	☒	A full description of the statistical parameters including central tendency (e.g. means) or other basic estimates (e.g. regression coefficient) AND variation (e.g. standard deviation) or associated estimates of uncertainty (e.g. confidence intervals)
☐	☒	For null hypothesis testing, the test statistic (e.g. *F*, *t*, *r*) with confidence intervals, effect sizes, degrees of freedom and *P* value noted<br>*Give P values as exact values whenever suitable.*
☒	☐	For Bayesian analysis, information on the choice of priors and Markov chain Monte Carlo settings
☒	☐	For hierarchical and complex designs, identification of the appropriate level for tests and full reporting of outcomes
☒	☐	Estimates of effect sizes (e.g. Cohen's *d*, Pearson's *r*), indicating how they were calculated

Our web collection on statistics for biologists contains articles on many of the points above.

Software and code

Policy information about availability of computer code

Data collection	No software was used for data collection
Data analysis	R, R base functions, DESeq2, bismark, bowtie2, ggplot2, deeptools, pheatmap, RColorBrewer, and several custom codes, available (public) on Github (https://github.com/perllb/DNMT1KO)

For manuscripts utilizing custom algorithms or software that are central to the research but not yet described in published literature, software must be made available to editors/reviewers. We strongly encourage code deposition in a community repository (e.g. GitHub). See the Nature Research guidelines for submitting code & software for further information.

Data

Policy information about availability of data

All manuscripts must include a data availability statement. This statement should provide the following information, where applicable:

- Accession codes, unique identifiers, or web links for publicly available datasets
- A list of figures that have associated raw data
- A description of any restrictions on data availability

- All sequencing data is deposited at the NCBI GEO: GSE107580.
- The mass spectrometry data is deposited at the ProteomeXchange Consortium: PXD008648.

- Figures with associated raw data:
Fig 1d-f
Fig 2a-c,e-j
Fig 3a-g
Supp Fig 1d,e,g,i-m
Supp Fig 3a-b

Supp Fig 4c-e
Supp Fig 5a
Supp Table 1

Field-specific reporting

Please select the one below that is the best fit for your research. If you are not sure, read the appropriate sections before making your selection.

☒ Life sciences ☐ Behavioural & social sciences ☐ Ecological, evolutionary & environmental sciences

For a reference copy of the document with all sections, see nature.com/documents/nr-reporting-summary-flat.pdf

Life sciences study design

All studies must disclose on these points even when the disclosure is negative.

Sample size	Sample size of n=3 for both the control and DNMT1-KO groups was chosen based on extensive experience of working with knock-out experiments in cell lines.
Data exclusions	No data was excluded from the analyses
Replication	The results were replicated repeatedly in two independent cell lines.
Randomization	This is not relevant to this study as it is performed in cell lines and includes one control group and one knock-out group.
Blinding	Blinding is not relevant for this study, as all cell quantification was automatically performed by using the Cellomics Scan 6.6.0 from Thermo Scientific and software from HCS Studio.

Reporting for specific materials, systems and methods

We require information from authors about some types of materials, experimental systems and methods used in many studies. Here, indicate whether each material, system or method listed is relevant to your study. If you are not sure if a list item applies to your research, read the appropriate section before selecting a response.

Materials & experimental systems

n/a	Involved in the study	
☐	☒	Antibodies
☐	☒	Eukaryotic cell lines
☒	☐	Palaeontology
☒	☐	Animals and other organisms
☒	☐	Human research participants
☒	☐	Clinical data

Methods

n/a	Involved in the study	
☐	☒	ChIP-seq
☐	☒	Flow cytometry
☒	☐	MRI-based neuroimaging

Antibodies

Antibodies used	All antibodies used in this study are routinely used in our lab:<br>- 5mC, Active Motif, cat.no. 39649, lot 06116002<br>- NESTIN, BD, cat.no 611658, lot 45333<br>- SOX2, R&D, cat.no MAB2018, lot KGQ0200041<br>- BrdU, Serotech, cat.no OBT0030, lot 0512<br>- L1 ORF1, Millipore, cat.no. MABC1152, lot 3137651
Validation	All antibodies used in this study are routinely used in our lab:<br>- We have previously validated the NESTIN and SOX2 antibodies in human embryonic tissue.<br>- BrdU stainings always included cells not exposed to BrdU as a negative control.<br>- The 5mC staining was validated in our NPC cultures by comparing control and DNMT1-KO cells (where 5mC was absent).<br>-The L1 ORF1p antibody is validated in literature

Eukaryotic cell lines

Policy information about cell lines

Cell line source(s)	The two cell lines were obtained from the Falk lab at Karolinska Institute, Stockholm, Sweden.

Authentication	We have received both cell lines at low passage number directly from the creator of these cell lines (Falk Lab, Karolinska Institute, Sweden). The cells lines were characterized as human NPCs by RNAseq, ICC and by differentiation into neurons.
Mycoplasma contamination	Both cell lines tested negative for mycoplasma.
Commonly misidentified lines (See ICLAC register)	No commonly misidentified cell lines were used.

ChIP-seq

Data deposition

☒ Confirm that both raw and final processed data have been deposited in a public database such as GEO.

☐ Confirm that you have deposited or provided access to graph files (e.g. BED files) for the called peaks.

Data access links<br>*May remain private before publication.*	NCBI GEO: GSE107580<br>Note: No peak calling were performed in this study, only raw fastq and bigWig files deposited
Files in database submission	GSM3557733 ChIP-seq_H3K27ac_CTR_1<br>GSM3557734 ChIP-seq_H3K27ac_CTR_2<br>GSM3557735 ChIP-seq_pol-II_CTR_1<br>GSM3557736 ChIP-seq_pol-II_CTR_2<br>GSM3557737 ChIP-seq_H3K27me3_CTR_1<br>GSM3557738 ChIP-seq_H3K27me3_CTR_2<br>GSM3557739 ChIP-seq_H3K27ac_DNMT1ko_1<br>GSM3557740 ChIP-seq_H3K27ac_DNMT1ko_2<br>GSM3557741 ChIP-seq_pol-II_DNMT1ko_1<br>GSM3557742 ChIP-seq_pol-II_DNMT1ko_2<br>GSM3557743 ChIP-seq_H3K27me3_DNMT1ko_1<br>GSM3557744 ChIP-seq_H3K27me3_DNMT1ko_2
Genome browser session (e.g. UCSC)	*Provide a link to an anonymized genome browser session for "Initial submission" and "Revised version" documents only, to enable peer review. Write "no longer applicable" for "Final submission" documents.*

Methodology

Replicates	Two replicates per ChIP reaction.
Sequencing depth	- Read length: 50bp single end.<br>- Raw Total Reads:<br>GSM3557733 ChIP-seq_H3K27ac_CTR_1: 39854157<br>GSM3557734 ChIP-seq_H3K27ac_CTR_2: 36852414<br>GSM3557735 ChIP-seq_pol-II_CTR_1: 50343460<br>GSM3557736 ChIP-seq_pol-II_CTR_2: 44904852<br>GSM3557737 ChIP-seq_H3K27me3_CTR_1: 39854157<br>GSM3557738 ChIP-seq_H3K27me3_CTR_2: 36852414<br>GSM3557739 ChIP-seq_H3K27ac_DNMT1ko_1: 42856848<br>GSM3557740 ChIP-seq_H3K27ac_DNMT1ko_2: 41418651<br>GSM3557741 ChIP-seq_pol-II_DNMT1ko_1: 42490145<br>GSM3557742 ChIP-seq_pol-II_DNMT1ko_2: 45449125<br>GSM3557743 ChIP-seq_H3K27me3_DNMT1ko_1: 31868284<br>GSM3557744 ChIP-seq_H3K27me3_DNMT1ko_2: 35009116<br>- Uniquely mapping reads:<br>GSM3557733 ChIP-seq_H3K27ac_CTR_1: 36968227<br>GSM3557734 ChIP-seq_H3K27ac_CTR_2: 34126276<br>GSM3557735 ChIP-seq_pol-II_CTR_1: 42537335<br>GSM3557736 ChIP-seq_pol-II_CTR_2: 38034627<br>GSM3557737 ChIP-seq_H3K27me3_CTR_1: 42537335<br>GSM3557738 ChIP-seq_H3K27me3_CTR_2: 38034627<br>GSM3557739 ChIP-seq_H3K27ac_DNMT1ko_1: 39597889<br>GSM3557740 ChIP-seq_H3K27ac_DNMT1ko_2: 38247264<br>GSM3557741 ChIP-seq_pol-II_DNMT1ko_1: 42537335<br>GSM3557742 ChIP-seq_pol-II_DNMT1ko_2: 38034627<br>GSM3557743 ChIP-seq_H3K27me3_DNMT1ko_1: 26388346<br>GSM3557744 ChIP-seq_H3K27me3_DNMT1ko_2: 29031017
Antibodies	- H3K27me3 (Millipore; cat# 07-449, lot# 3018864)<br>- H3K27Ac (Diagenode, cat# C15410196, lot# A1723-0041D)<br>- Pol II (RPB2, N-term; GeneTex, cat# GTX102535-S, lot# 39918)
Peak calling parameters	No peak calling performed

Data quality	Read quality assessed with fastqc. Low quality reads discarded. Quality of ChIP-experiment assessed deeptools analysis and quality control, as well as by inspection in UCSC, for positive control targets.
Software	bowtie2 for mapping, bam/sam/bedtools used for processing of mapped reads, and deeptools for visualization and analysis.

Flow Cytometry

Plots

Confirm that:

☒ The axis labels state the marker and fluorochrome used (e.g. CD4-FITC).

☒ The axis scales are clearly visible. Include numbers along axes only for bottom left plot of group (a 'group' is an analysis of identical markers).

☒ All plots are contour plots with outliers or pseudocolor plots.

☒ A numerical value for number of cells or percentage (with statistics) is provided.

Methodology

Sample preparation	The cells were de-attached, resuspended in media containing PI for dead cell exclusion and filtered with a 70um filter.
Instrument	BD Biosciences FACS Aria III
Software	Diva 8.0.1
Cell population abundance	A minimum of 200 cells were sorted into a tube containing PBS and PI. The sorted cells were reanalyzed and the % GFP positive cells out of live cells was determined and always fell within 98-100%.
Gating strategy	Live cells were gated for using PI. The GFP positive gate was set based on untransduced cells vs GFP expressing cells. The GFP+ cells were validated to be the correct population by both ICC and sequencing.

☒ Tick this box to confirm that a figure exemplifying the gating strategy is provided in the Supplementary Information.
